# Case of Constipation, with Vomiting

**Published:** 1853-05

**Authors:** Henry Yale Smith


From the Philadelphia Medical and Surgical Journal.

*A Case of Constipation, with vomiting, &c.* By HENRY YALE SMITH, M.D.

ON the 17th of June, 1851, I was visited at my office by Mr. H., aged 35 years; he gave me the history and symptoms of his case as follows:—In the early part of the night previous he began to suffer from pain in the abdomen, which progressively increased in severity, with occasional remissions, until 9 o'clock A.M., when he deemed it proper to call on me professionally. I then ascertained that his bowels had not been moved for four days prior to the attack, for which he had taken a gentle aperient. Finding it had not operated, four Comp. Cath. Pil. were ordered, with the understanding, should the bowels not be opened in two hours, to have administered the enema of castor oil and turpentine. At 2 o'clock P. M. a messenger was despatched requesting my attendance. On obeying the summons, I found the pain increased. Quarter of a grain of sulphate of morphia every half hour for two hours, with sinapisms to the abdomen, was ordered The pain was in some measure alleviated, until 11 o'clock, but from that time continued up to my visit the next morning at 8 o'clock. I then covered his abdomen with fresh sinapisms, gave an enema of turpentine with gruel; after which XX grains of calomel were given him, followed by 8 gr. ij. of Croton oil made into pills. He took during the morning two grs. of sulph. morphiæ. Occasionally he vomited, but this was not a prominent symptom and seldom occurred, save when fluids of medicine were taken into the stomach; that which was thrown off was a turbid, mucous fluid. Frequently he would rise to his chair with the view of easing himself; at the same time would press down, but to no purpose, nor did any flatulence or wind come from the bowels. 11½ o'clock A. M. a bottle of citrate of magnesia was given, also an enema of soap, ol. ricini and turpentine. 3 P. M., eight wet cups and four dry cups were applied over the seat of pain, followed up by warm fomentations, &c. The relief obtained from the cups was but temporary, as the pain returned in a few hours. I again visited him at 8 o'clock P. M. and found him as bad as ever—the abdomen was universally tender on pressure, but not so much as prior to the cupping. He was evidently much prostrated, skin warm, tongue furred and dry, pulse quick and wirey—about 120. Fearing to give any more cathartic medicine, I left him, saying that I would return again at 11 o'clock that evening, at which time I did, armed with a flexible tube. The patient was placed on his hands and knees, and the tube was passed into the rectum with considerable difficulty, being detained several times by obstructions, which yielded to gentle pressure. About a pint to a pint and a half of lukewarm water, with two ounces of lamp oil added, was injected through the tube into the intestines, when he complained of a sense of distension and an irresistible desire to evacuate the bowels. When the syringe was removed from the tube, a portion of the injected fluid came away through the tube, containing numerous small flocculi of mucus tinged with fœcal matter. About five minutes after removing the stomach tube, he passed about half a gallon of the injected fluid, containing solid fœces of a dark brown color, from the size of a hickory nut to that of a walnut, which occurred with considerable pain; two subsequent evacuations occurred through the night, with little or no pain. On visiting my patient the following morning, I found he had rested tolerably well between three and six in the morning; his pulse was about 95, abdomen slightly tympanitic, still tender on pressure; being much prostated, he complained chiefly of a sense of weakness, for which the ordinary remedies were used.

